# Effectiveness of Transcutaneous Bilirubin Measurement in High-Risk Neonates and to Evaluate Validity of Transcutaneous Bilirubin With Total Serum Bilirubin Levels in Both Low and High-Risk Neonates at a Tertiary Care Center in a Developing Country

**DOI:** 10.7759/cureus.13685

**Published:** 2021-03-04

**Authors:** Durre Shahwar Khan, Adnan Mirza, Areesh Bhatti, Ali Shabbir, Batha Tariq, Arjumand Rizvi

**Affiliations:** 1 Neonatology, Liaquat National Hospital, Karachi, PAK; 2 Paediatric - Neonatal Medicine, Limerick University Maternity Hospital, Limerick, IRL; 3 Neonatal Intensive Care, Aga Khan University Hospital, Karachi, PAK; 4 Pediatric Medicine, Aga Khan University Hospital, Karachi, PAK; 5 Neonatology, Aga Khan University Hospital, Karachi, PAK; 6 Research and Development, Aga Khan University Hospital, Karachi, PAK

**Keywords:** high-risk neonates, hyperbilirubinemia, transcutaneous bilirubin (tcbr), total serum bilirubin (tsbr)

## Abstract

Objectives

To evaluate the usefulness of transcutaneous bilirubin (TcBR) nomogram in high-risk neonates and to identify the validity of TcBR and total serum bilirubin (TsBR) in both low and high-risk neonates to guide management in under-resourced settings.

Methodology

A cross-sectional study was conducted at the well-baby nursery of a tertiary care center in Karachi, Pakistan. All neonates admitted in the well-baby nursery with jaundice were stratified into high and low-risk groups. Eighty-seven neonates were included in the low-risk group and 121 neonates in the high-risk group. The usefulness of the TcBR nomogram in high-risk neonates and the validity of TcBR and TsBR in both low and high-risk neonates were determined through sensitivity and specificity analysis.

Results

The correlation coefficients (r) were found to be comparable in the high-risk group (r = 0.82, p < 0.001) and the low-risk group (r = 0.87, p < 0.001). The specificity of cutaneous bilirubin measurement based on bilirubin levels in the high-risk group was higher (93.0%) than that of the low-risk group (90.1%). However, the sensitivity was found to be lower (60.0%) in the high-risk group compared to the low-risk group (68.8%). The mean value of TsBR was equal in both groups. The mean TcBR in the high-risk group was 10 ± 2.3 compared to 11 ± 2.1 in the low-risk group. Phototherapy was given in 67.0% of the high-risk cases and 41.4% of the low-risk cases. Bland Altman analysis was also performed to depict the relationship between TcBR and TsBR measurements.

Conclusion

The TcBR nomogram was effective in high-risk neonates and also had validity in both high and low-risk neonates. A phototherapy-driven protocol based on TcBR would be a cost-effective and useful tool in the identification and management of neonatal jaundice in both high and low-risk groups in developing countries like Pakistan.

## Introduction

Neonatal hyperbilirubinemia is one of the most common problems encountered in pediatric clinical practice and remains an issue of immense interest and discussion [1]. Hyperbilirubinemia is defined as a total serum bilirubin (TsBR) level > 95th percentile on the hour-specific nomogram [2]. An increase in TsBR can manifest as neonatal jaundice, which is a yellow discoloration of the skin and/or sclera caused by bilirubin deposition. Worldwide, 60% of term and 80% of preterm neonates develop jaundice [3-4]. Thus, neonatal jaundice is one of the most common causes of hospital admissions in neonates, particularly in Asia [5-6], and its timely identification and treatment is crucial. If left untreated, high bilirubin levels can result in serious complications, including acute bilirubin encephalopathy and chronic bilirubin-induced neurologic dysfunction (BIND), often leading to permanent and irreversible consequences for the child [7].

Serum bilirubin measurement is currently the gold standard for the evaluation of neonatal jaundice. However, it involves invasive and painful venipunctures, in addition to being time-consuming and resource-intensive [8-10]. In Pakistan, the major mode of healthcare financing is out-of-pocket, and the healthcare system is significantly strained. Therefore, the search for a low-cost method for routine neonatal screening is highly pertinent. Transcutaneous bilirubinometry (TcBR), first introduced in 1980, is increasingly being used as an alternative, cost-effective screening tool for the measurement of bilirubin concentration in neonates and is well-established in the developed world [11]. It employs the use of skin remittance spectra analysis, wherein skin is exposed to light and the returning light is analyzed. The spectra of returning light are influenced by the presence of bilirubin in the skin, which the device then uses to calculate the concentration of bilirubin [12]. The use of this method can rectify the need for invasive venipunctures and TsBR measurement. Transcutaneous bilirubinometry has proven to be a reliable screening tool for hyperbilirubinemia in both term and preterm neonates [13]. In a study by Sanpavat et al., it was shown that screening with TcBR would eliminate painful venipunctures done on premature neonates by 40%, thus avoiding its numerous adverse effects [2].

Studies have shown a strong correlation between TcBR and TsBR measurements, indicating that TcBR is a reliable screening tool. A screening protocol utilizing the TcBR nomogram for low-risk neonates has been published from our center with promising results [13-14]. However, to the best of our knowledge, no study has been carried out to investigate the utility of the TcBR nomogram in Pakistan for high-risk neonates. Therefore, the aim of our study is to evaluate the utility of TcBR as a screening tool in high-risk neonates. If the TcBR measurements are found to be reliable in our population, it could significantly reduce the frequency of invasive blood sampling in high-risk neonates and help reduce costs. We also aim to assess the validity of TcBR and TsBR in both high and low-risk neonates.

## Materials and methods

A cross-sectional study was carried out from May 1, 2019 to October 31, 2019 at the Aga Khan University Hospital (AKUH), Karachi. AKUH is a tertiary care center located in a highly populated urban area of Karachi, Sindh. At our center, around 5,500 neonates are delivered every year, out of which about 85% are term and 15% are preterm births. There are two postnatal well-baby nurseries with a combined capacity of 120 patients. Currently, all neonates admitted in the well-baby nurseries are assessed by a neonatologist, fellow, resident, and nursing staff.

Neonates admitted to the well-baby nursery with jaundice were included in the study. Neonates with a gestational age of < 35 weeks, neonates requiring neonatal intensive care unit (NICU) admission, neonates who were on phototherapy or had received phototherapy in the past, neonates with conjugated hyperbilirubinemia, and neonates who developed clinical jaundice after seven days of life were excluded. All healthy neonates with a gestational age of 37 weeks or above, birth weight of 2.5 kg or above, and no risk factors associated with the development of significant jaundice were classified as low-risk babies. Neonates with a gestational age of 35 weeks to 36 + 6 weeks, rhesus isoimmunization, ABO isoimmunization, a family history of glucose-6-phosphate dehydrogenase (G6PD) deficiency, jaundice within the first 24 hours of life, a history of neonatal jaundice in siblings treated with phototherapy/exchange transfusion, or a history of siblings who developed kernicterus were classified as high-risk babies. Overall, 87 low-risk babies and 121 high-risk babies were included in this study.

Basic demographic and anthropometric data were recorded, including gestational age, mother’s blood group, baby’s blood group, and other risk factors for jaundice. Two Dräger JM-105 bilirubinometers (Drägerwerk AG & Co., Lübeck, Germany) were used for monitoring TcBR, one for each well-baby nursery. Three readings were taken from the sternum. The mean of three readings was calculated, recorded, and plotted on the TcBR nomogram for low-risk and high-risk neonates separately.

Blood sampling for TsBR, when indicated, was done via venipuncture by the nurse in the well-baby nursery or a phlebotomist, as per protocol, and samples were sent to the laboratory for analysis. The training was carried out for competency certification of all neonatal healthcare providers involved. The content of training included stratification of newborns to high and low-risk, familiarization and plotting of the nomogram, and usage of transcutaneous bilirubinometers. The protocol flow chart and TcBR nomogram were handed over to all postnatal nurses and physicians and pasted in all postnatal ward areas for reference. Descriptive analysis was performed using the Statistical Package for Social Sciences (SPSS), version 19, (IBM SPSS Statistics for Windows, Armonk, NY) using the mean and standard deviation for normally distributed and the median and interquartile range (IQR) for non-normally distributed continuous variables. Frequency and percentages were reported for categorical variables. Bland Altman plots were constructed to depict the relationship between TcBR and TsBR measurements. Correlation coefficients were calculated using Pearson correlation and the sensitivity and specificity of TcBR and TsBR measurements. The analysis was performed separately for high-risk and low-risk infants to assess the validity of TcBR independently in both groups.

For neonates who developed jaundice within 24 hours of birth, TsBR was sent and the jaundice was managed according to American Academy of Pediatrics (AAP) guidelines [15]. If neonates developed jaundice after 24 hours, risk assessment was performed.

**Figure 1:**
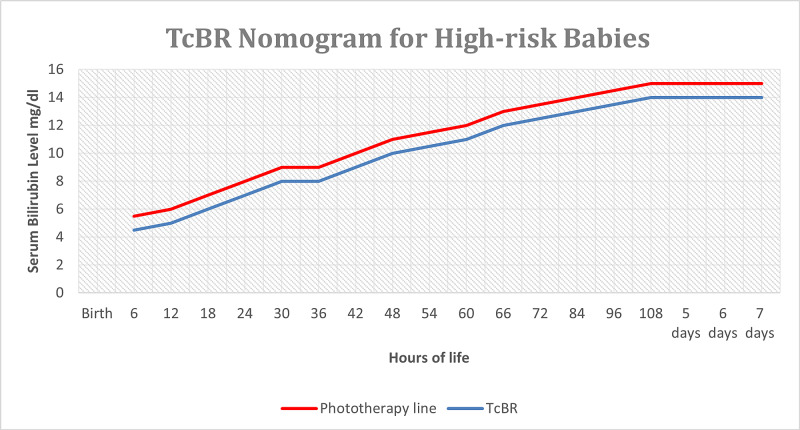
Transcutaneous bilirubinometry (TcBR) nomogram for high-risk neonates

The TcBR nomogram for high-risk neonates was made using the AAP guidelines as a reference for the phototherapy threshold (red line, Figure 1) [16]. A new line was drawn 1 mg/dL (34.2 µmol/L) below the phototherapy line (red line) for high-risk babies and named as TcBR line (blue line) since the literature review reveals a variation of ± 1 mg/dL (17.1 µmol/L) in the results of TcBR and TsBR. For simplification, the intermediate and low-risk lines are removed from the chart since those neonates are not a part of the study population, and their management is done according to the hospital’s jaundice protocol. The lines are color-coded. The phototherapy line is in red, whereas the TcBR line is in blue. For simplification, the low and intermediate-risk lines are removed from the AAP nomogram. This modification in the AAP nomogram is the high-risk TcBR nomogram.

For high-risk babies, upon the appearance of clinical jaundice, consent was taken for TcBR testing in a pre-designed consent form. The study was explained to the parents of babies who fulfilled the eligibility criteria by the healthcare provider. TcBR was then plotted on the TcBR nomogram for high-risk babies. If the TcBR level fell on or above the red line, the TsBR was sent and a second TcBR was done within 30 minutes for validation purposes. An effort was made to repeat the TcBR within 30 minutes for validation purposes. However, if it was delayed, we excluded the baby from the study.

If the TcBR level fell on the blue line, then TsBR was sent, another TcBR was done within 30 minutes for validation; phototherapy was started only if TsBR fell on or above the red line. All babies with a TcBR level below the blue line were followed with TcBR every eight hours until a resolution of clinical jaundice or discharge. The values of TsBR and TcBR were recorded in a pre-designed proforma for validation.

**Figure 2:**
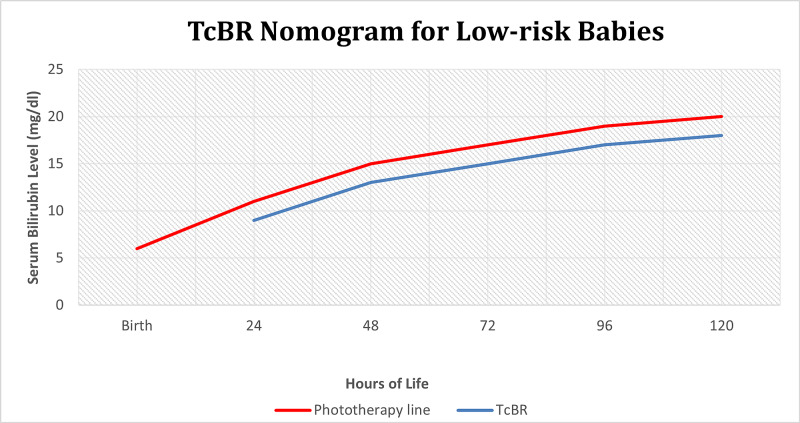
Transcutaneous bilirubinometry (TcBR) nomogram for low-risk neonates

For neonates categorized as low-risk, the TcBR was checked and plotted on the TcBR nomogram for low-risk neonates (Figure 2) [14, 16]. The TcBR nomogram for low-risk neonates was already in use at our institute and was created using the AAP guidelines for phototherapy threshold for low-risk babies. It was incorporated into the hospital’s guidelines for the management of neonatal jaundice. It is similar to the high-risk nomogram and also has two color-coded lines, a red line (phototherapy line) which shows the phototherapy threshold for low-risk babies, and a blue line (TcBR line) drawn 2 mg/dL (34.2 µmol/L) below the phototherapy line and is named as the TcBR line because a literature review reveals a variation of ± 1 mg/dL (17.1 µmol/L) in results of TcBR and TsBR. For simplification, the high and intermediate-risk lines are removed from the AAP nomogram. This modification in the AAP nomogram is called TcBR nomogram for low-risk neonates.

For all low-risk babies, the appearance of clinical jaundice TcBR was checked and plotted on the TcBR nomogram for low-risk neonates [14, 16]. If the TcBR level fell on or above the red line, TsBR was sent (according to the departmental protocol in practice already), a repeat TcBR was done within 30 minutes for validation, and phototherapy is started. If the TcBR level fell on the blue line, then TsBR was sent, TcBR was done within 30 minutes for validation, and phototherapy was started only if the TsBR fell on or above the red line. All babies with a TcBR level below the blue line were followed with a TcBR every eight hours until a resolution of clinical jaundice or discharge. The values of TsBR and TcBR were recorded in a pre-designed proforma for validation.

## Results

Overall, 121 high-risk neonates and 87 low-risk neonates were included in this cross-sectional study. Of the high-risk neonates, 69 (57.0%) were male and 52 (43.0%) were female. Of the low-risk neonates, 46 (53.0%) were male and 41 (47.0%) were female. The median postnatal age was 50 hours in both groups. The mean gestational age was slightly lower in the high-risk group, i.e., 37.0 ± 1.0 weeks compared to the low-risk group, i.e., 38 ± 0.4 weeks. The mean birth weight of the neonates was also lower in the high-risk group (2.6 ± 0.5 kg) compared to the low-risk group (3.1 ± 0.4 kg). The median onset of jaundice was sooner at 30 hours in high-risk neonates compared to 33 hours in low-risk neonates. In the high-risk group, four children had a history of jaundice in siblings requiring treatment with phototherapy, one child had a history of kernicterus in siblings, and two children had a family history of G6PD deficiency (Table 1).

**Table 1:** Characteristics of the Study Population G6PD: glucose-6-phosphate dehydrogenase; IQR: interquartile range; SD: standard deviation

Characteristics	High-risk group (n = 121)	Low-risk group (n = 87)
Male gender	69 (57.0%)	46 (53.0%)
Postnatal age in hours; median (IQR)	50 (41 - 50)	50 (45 - 50)
Gestational age in weeks; median (IQR)	37 ± 1.9	38 ± 0.4
Birth weight in kg; mean ± SD	2.6 ± 0.5	3.1 ± 0.4
Height in cm; median (IQR)	49 (45 - 50)	50 (48 - 50)
Onset of jaundice in hours of life; median (IQR)	30 (24 - 37)	33 (28 - 40)
History of jaundice in sibling(s) requiring treatment with phototherapy	4 (3.3%)	-
History of kernicterus in sibling(s)	1 (0.8%)	-
History of G6PD deficiency in the family	2 (1.7%)	-

Based on our results, the mean value of TsBR was equal in both groups (Table 2). However, the mean TsBR time in the high-risk group was 35 ± 13 hours compared to 38 ± 9.7 hours in the low-risk group. The mean TcBR in the high-risk group was 10 ± 2.3 compared to 11 ± 2.1 in the low-risk group. The mean TcBR within 30 minutes was 10 ± 2.3 in the high-risk group compared to 10 ± 1.9 in the low-risk group.

**Table 2:** TcBR and TsBR Measurements, Number of Times Measured, Phototherapy Initiation, and Phototherapy Initiation Time IQR: interquartile range; SD: standard deviation; TcBR: transcutaneous bilirubinometry; TsBR: total serum bilirubin

TcBR and TsBR	High-risk neonates	Low-risk neonates
TsBR value (mean ± SD)	10 ± 2.5	10 ± 2.0
TsBR time in hours (mean ± SD)	35 ± 13	38 ± 9.7
TcBR value (mean ± SD)	10 ± 2.3	11 ± 2.1
TcBR within 30 minutes (mean ± SD)	10 ± 2.3	10 ± 1.9
Number of times TsBR done		
1. One time	49 (41.2%)	68 (78.0%)
2. Two times	26 (21.8%)	10 (11.0%)
3. Three times	39 (32.8%)	9 (10.0%)
4. More than three times	7 (5.2%)	0 (00.0%)
Number of times TcBR done		
1. One time	35 (28.9%)	0 (00.0%)
2. Two times	26 (21.5%)	0 (00.0%)
3. Three times	47 (38.8%)	87 (100.0%)
4. More than three times	13 (10.8%)	0 (00.0%)
Phototherapy initiated based on TcBR	81 (67.0%)	36 (41.4%)
Phototherapy initiation time in hours; median (IQR)	34 (25-46)	34 (30-41)

In 78% of the low-risk neonates, TsBR was sent only once, whereas in 59.8% of the high-risk neonates, the TsBR was sent two or more times. In the low-risk group, TcBR was done three times in all the cases, whereas TcBR was done three times in only 38.8% cases of the high-risk group. Based on TcBR results, phototherapy was initiated in 67.0% of cases in the high-risk group, whereas it was started in only 41.4% of cases in the low-risk group. However, the median time of initiation of phototherapy was 34 hours in both groups (Table 2).

**Figure 3:**
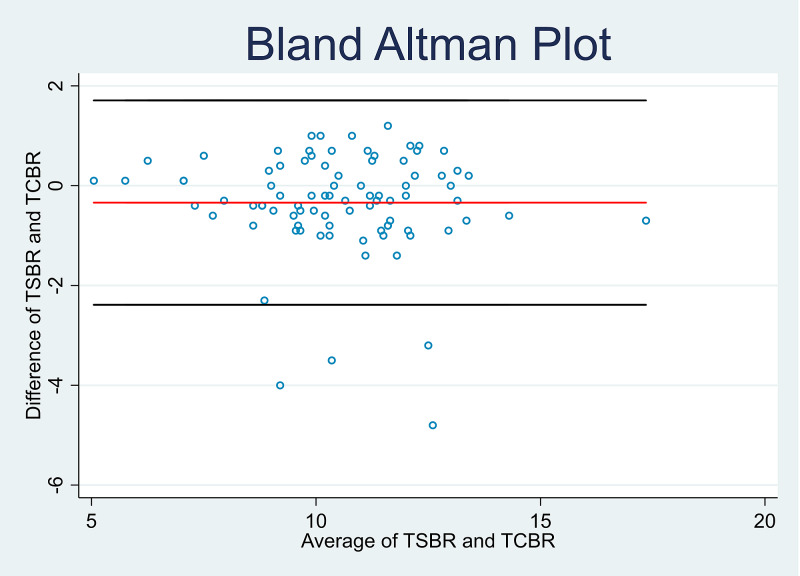
Bland Altman plot for low-risk babies TcBR: transcutaneous bilirubinometry; TsBR: total serum bilirubin

**Figure 4:**
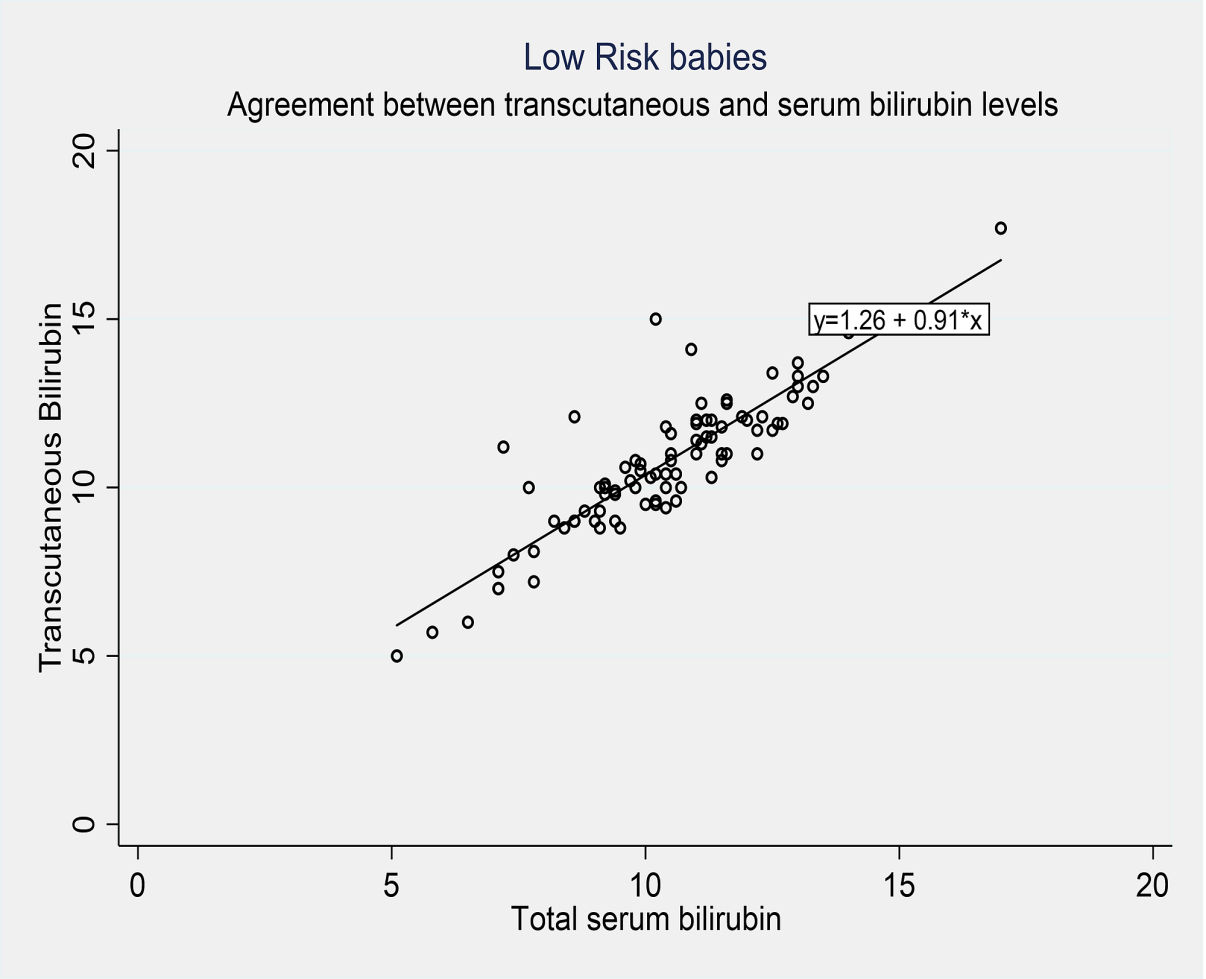
Pearson’s correlation scatter plot for low-risk babies

**Figure 5:**
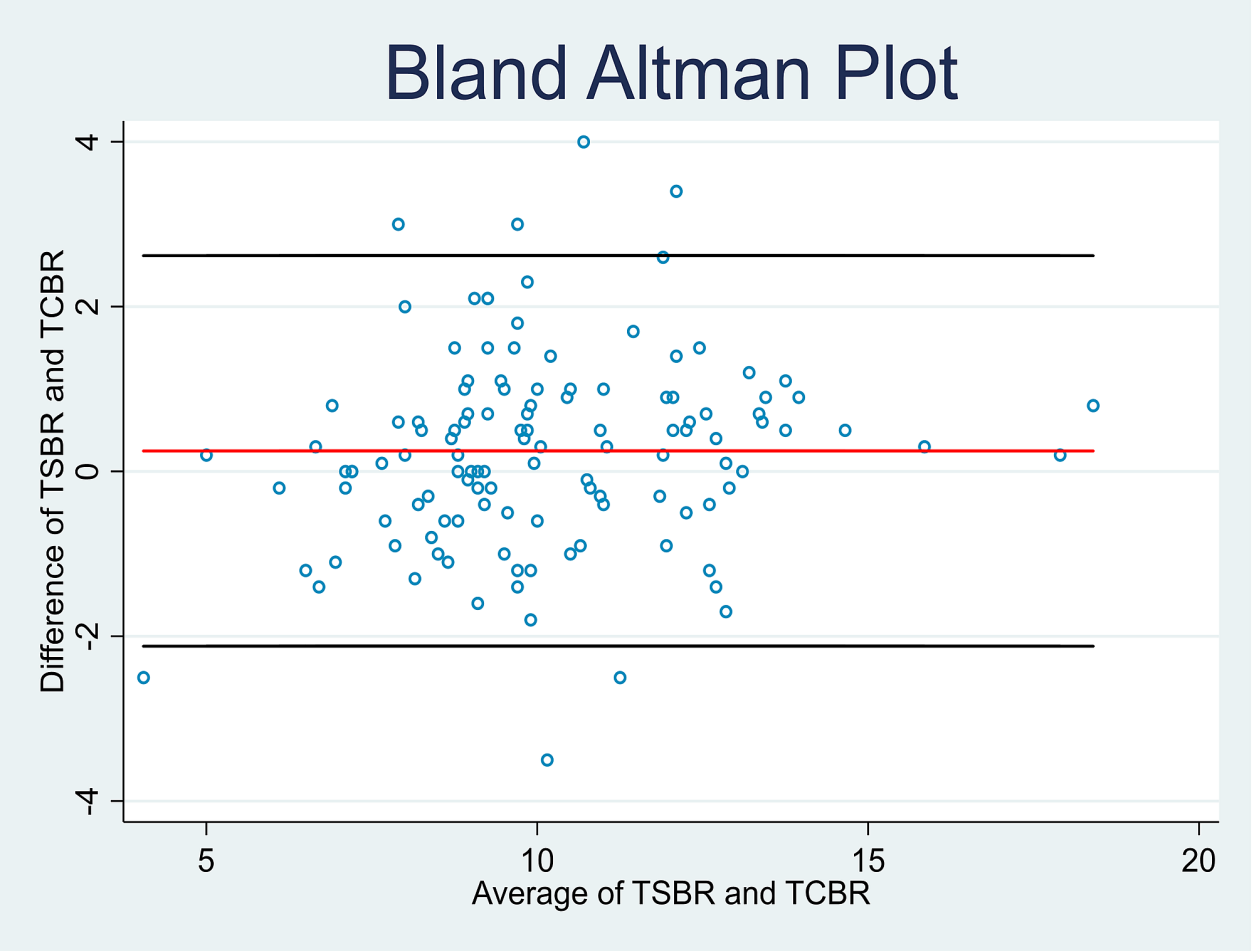
Bland Altman plot for high-risk babies TcBR: transcutaneous bilirubinometry; TsBR: total serum bilirubin

**Figure 6:**
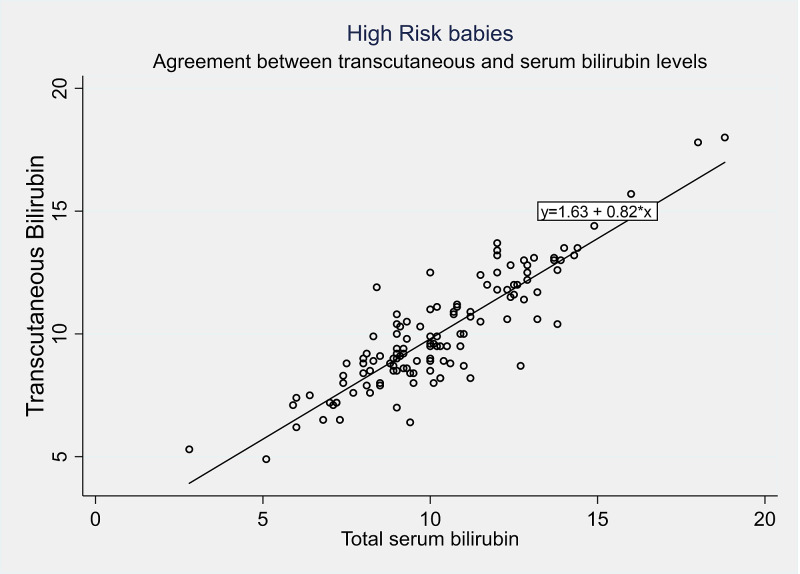
Pearson’s correlation scatter plot for high-risk babies

Further analysis was done to determine the correlation coefficient, sensitivity, and specificity of the cutaneous bilirubin measurement (TcBR) and serum bilirubin (TsBR) levels in both high and low-risk babies. The correlation coefficients were similar in the high-risk group (r = 0.82, p < 0.001) and the low-risk group (r = 0.87, p < 0.001). The specificity of the cutaneous bilirubin measurement based on serum bilirubin levels in the high-risk group was 93.0% compared to 90.1% in the low-risk group. However, the sensitivity was found to be lower at 60.0% in the high-risk group compared to 68.8% in the low-risk group. Bland Altman plots were also constructed to depict the relationship between TcBR and TsBR measurements (Figures 3-4). Correlation coefficients were calculated using the Pearson correlation to determine the sensitivity and specificity of TcBR measurements (Tables 3-4, Figures 5-6).

**Table 3:** Correlation, Sensitivity, and Specificity of the TcBR Measurement and TsBR Levels (TsBR ≥ 12 mg/dL is Abnormal) SD: standard deviation; TcBR: transcutaneous bilirubinometry; TsBR: total serum bilirubin

	High-risk babies	Low-risk babies
Linear regression and Spearman rank correlation coefficients for TcBR with TsBR	r = 0.82	r = 0.87
	P < 0.001	P < 0.001
Specificity and sensitivity of cutaneous bilirubin measurement based on serum bilirubin levels	At bilirubin level ≥ 12 mg/dL, sensitivity = 60.7%	At bilirubin level ≥ 12 mg/dL, sensitivity = 68.8%
	Specificity = 93.5%	Specificity = 90.1%
Frequency and percentage of abnormal serum bilirubin versus cutaneous bilirubin	TcBR abnormal = 23 (19.0%)	TcBR abnormal = 18 (20.7%)
	TsBR abnormal = 28 (23.1%)	TsBR abnormal = 16 (18.4%)
	Both abnormal = 17	Both abnormal = 11
	Only TcBR abnormal = 6	Only TcBR abnormal = 7
	Only TsBR abnormal = 11	Only TsBR abnormal = 5

**Table 4:** Mean ± SD Cutaneous Bilirubin Levels Based on Age, Sensitivity, and Specificity SD: standard deviation

High-Risk Babies		Low-Risk Babies	
First day of life (n: 9): sensitivity	60.0%	First day of life: sensitivity	00.0%
Specificity	100.0%	Specificity	00.0%
Second day of life (n: 43): sensitivity	50.0%	Second day of life (n: 38): sensitivity	100.0%
Specificity	97.3%	Specificity	88.2%
Third day of life (n: 69): sensitivity	64.7%	Third day of life (n: 47): sensitivity	58.3%
Specificity	90.4%	Specificity	91.4%
Total (n: 121): sensitivity	60.7%	Total (n: 87): sensitivity	68.8%
Specificity	93.5%	Specificity	90.0%

## Discussion

The results of our study indicate a highly positive correlation between TcBR and TsBR measurements, which supports the use of TcBR measurement as a reliable screening tool for high-risk neonates, along with the use of appropriate clinical judgment.

Jaundice in neonates is a common cause of hospitalization in the first 30 days of life, and complications related to hyperbilirubinemia are severe but preventable. For this reason, an accurate evaluation of every newborn for hyperbilirubinemia is crucial. However, it comes at the cost of repeated venipunctures resulting in pain and other complications, as well as an increased financial cost which can be a very significant issue in low and middle-income countries, such as Pakistan. This is why the search for a non-invasive, low-cost method is important globally and even more so in our part of the world.

According to our data, there was a high positive correlation (r = 0.82, p < 0.001) between TcBR and TsBR values for high-risk neonates. This indicates that the values are comparable and TcBR is a reliable enough substitute for TsBR in high-risk neonates. This correlation was stronger (r = 0.87, p < 0.001) in the low-risk group. Similarly, our study found the sensitivity of cutaneous bilirubin measurements based on serum bilirubin levels at a bilirubin level ≥ 12 mg/dL to be 60.7% for the high-risk group, lower than that of the low-risk group (68.8%), indicating that TcBR might be better suited for low-risk neonates. This sensitivity of 60.7% calls into question the utility of this test as a screening tool, but it must be weighed against the potential harm of an invasive screening test, i.e., TsBR. We suggest that TcBR is used as a screening tool in conjunction with appropriate clinical judgment, considering its limited sensitivity. The specificity, on the other hand, was high in both groups but slightly lower in the low-risk group (90.1%) compared to the high-risk group (93.5%).

Based on the findings of our study, TcBR can be regularly employed as a screening tool for high-risk neonates instead of the invasive blood sampling for TsBR. This can help prevent unnecessary venipunctures in neonates and cut costs for families and healthcare systems. However, this should be employed mainly as a screening tool. For evaluation and decision-making regarding the treatment of neonates found to have hyperbilirubinemia, a confirmatory TsBR should be considered. The results of a TcBR test should be viewed in conjunction with other variables, such as the patient’s clinical examination, risk status, etc. The attending physician’s clinical judgment of a child’s condition and risk-status must always play a role and physicians might choose to directly check TsBR where indicated.

Our data shows that, at our center, TcBR values were useful in guiding decision-making regarding the initiation of phototherapy in low-risk and high-risk neonates. Sixty-seven percent of the high-risk babies were put on phototherapy based on their TcBR results. This proves that TcBR can help facilitate quicker and easier decision-making for neonates with hyperbilirubinemia. Employing the use of TcBR might have also reduced the number of unnecessary venipunctures for TsBR, as very few of the neonates had to get repeated venipunctures in the low-risk group. Most (78.0%) of neonates in the low-risk group had to get only one TsBR measurement. In the high-risk group, 41.2% of neonates got only one TsBR measurement and were free from further venipunctures. The rest (58.8%), however, had to get a TsBR done at least twice.

A previously done study from our center on the implementation of TcBR protocols in low-risk neonates reported crude cost savings of $1,800 in six months which supports the idea that the use of TcBR as a screening tool can significantly reduce costs for patients and centers [14]. This protocol can be particularly useful in low-resource centers that cater to most of the population of our country as it can increase access to healthcare and appropriate screening while significantly reducing costs.

Similar studies that have been done in the region have shown promising results [17-19]. A 2015 study from Iran found a similar correlation coefficient of 0.82 between TcBR and TsBR measurements; however, they reported a higher sensitivity of 84.4% and a lower specificity of 84.2% [17]. Another study from Bangladesh published in 2017 found a stronger correlation between TcBR and TsBR measurements (r = 0.93, p < 0.001) [18]. A study from India done on preterm low-birth-weight neonates also found a good correlation between TcBR and TsBR measurements (r = 0.84, mean difference, 0.3 ± 1.9 mg/dL) [19].

Our study came from an urban tertiary care center with a diverse patient population, and we were able to obtain a decent sample size. However, as our study was cross-sectional, we were not able to follow our patients over time. Future studies should be done prospectively or retrospectively to observe how these neonates do over time in order to create appropriate guidelines for the use of TcBR as a screening tool in high-risk neonates. TcBR and TsBR should also be followed in patients receiving phototherapy to observe if this weakens the correlation between the two. Future studies should also look closely at the cost differences when using this protocol as opposed to using TsBR, as well as the reduction in the number of venipunctures and/or adverse effects of repeated venipunctures.

In conclusion, our study supports the use of TcBR as a cost-effective, non-invasive screening tool for high-risk neonates in low-resource settings, along with appropriate clinical evaluation and follow-up.

## Conclusions

According to our study results, we concluded that the TcBR nomogram is useful in ascertaining bilirubin levels in high-risk neonates and is valid for both low and high-risk neonates. A phototherapy guideline based on the TcBR nomogram could prove to be a cost-effective and convenient tool for the identification and management of neonatal jaundice, particularly in low-resource settings such as Pakistan.
